# Recruitment Challenges in Spinal Cord Stimulation Trial for Motor Recovery in Patients with Chronic Complete Spinal Cord Injury

**DOI:** 10.3390/jcm14113925

**Published:** 2025-06-03

**Authors:** Fatimah Misbaah, Wen Li Lui, Zhi Yan Valerie Ng, Seng Kwee Wee, Min Wee Phua, Rosa Q. So, Brian Premchand, Kezia Susanto, Seyed Ehsan Saffari, Rui Xin Justin Ker, Wai Hoe Ng, Kai Rui Wan

**Affiliations:** 1 Department of Neurosurgery, National Neuroscience Institute, Tan Tock Seng Hospital, Singapore 308433, Singapore; justin.ker.r.x@singhealth.com.sg (R.X.J.K.); ng.wai.hoe@singhealth.com.sg (W.H.N.); wan.kai.rui@singhealth.com.sg (K.R.W.); 2 Department of Neurosurgery, National Neuroscience Institute, Singapore General Hospital, Singapore 169608, Singapore; 3 Department of Rehabilitation Medicine, Tan Tock Seng Hospital, Singapore 308433, Singapore; wen_li_lui@ttsh.com.sg (W.L.L.); valerie_ng@ttsh.com.sg (Z.Y.V.N.); seng_kwee_wee@ttsh.com.sg (S.K.W.); min_wee_phua@ttsh.com.sg (M.W.P.); 4 Singapore Institute of Technology, Health and Social Sciences Cluter, Singapore 138683, Singapore; 5 Institute for Infocomm Research, Agency for Science, Technology and Research (A*STAR), Singapore 138632, Singapore; rosa-so@i2r.a-star.edu.sg (R.Q.S.); brian_premchand@i2r.a-star.edu.sg (B.P.); stuks@i2r.a-star.edu.sg (K.S.); 6 Department of Biomedical Engineering, National University of Singapore, Singapore 117583, Singapore; 7 Center for Quantitative Medicine, Duke-NUS Medical School, National University of Singapore, Singapore 119077, Singapore; gmsses@nus.edu.sg

**Keywords:** spinal cord injury, epidural spinal stimulation, neurosurgery, recruitment challenges, eligibility criteria, patient apprehension, neurorehabilitation, clinical research coordinator, research ethics

## Abstract

Recruiting participants for clinical trials targeting specific populations, like patients with chronic motor complete spinal cord injuries (SCIs), is challenging. The RESTORES trial evaluated spinal cord stimulation (SCS) combined with robotic neurorehabilitation for motor recovery in this population. This feasibility study enrolled three participants to assess SCS implant safety, synergistic effects of SCS and robotic rehabilitation, and clinical outcomes. Key recruitment barriers included the small patient pool, stringent eligibility criteria, patient skepticism, and logistical and emotional challenges. Strategies to address these challenges encompassed multidisciplinary collaborations with clinical centers, SCI associations, and patient support groups, including pre-surgical counselling and transparent communication. A dedicated clinical research coordinator ensured ethical compliance, logistical support, and participant engagement. Travel reimbursements, family involvement, and peer advocacy fostered accessibility and trust. Of the 115 patients screened, only 3 met the strict eligibility criteria, due to high screening failure rates and participant apprehension. Peer testimonials and family support helped enhance motivation and adherence. Ethical safeguards, like a data safety monitoring board, ensured participant safety and transparency. The RESTORES trial underscores the complexity of recruiting for pioneering interventions while highlighting the importance of tailored, patient-centric strategies. Insights gained will inform future trials and contribute to advancing SCI rehabilitation, offering hope for enhanced neurological recovery and quality of life for individuals with chronic motor complete SCI.

## 1. Introduction

Recruitment for clinical trials is a critical yet often challenging aspect of trial success, particularly when the target population is highly specific (such as patients with chronic spinal cord injuries (SCIs)), and the burden of participation is substantial for both the patient and their caregivers. In our RESTORES (RESToration Of Rehabilitative function with Epidural spinal Stimulation) trial [1], which focuses on combining spinal cord stimulation (SCS) with advanced robotic neurorehabilitation for motor recovery, recruitment challenges are amplified by the complexity of the condition, patient hesitancy, logistical barriers, and stringent eligibility criteria.

During the RESTORES trial, we hypothesized that combining SCS with advanced robotic neurorehabilitation can improve neurological function in patients with chronic motor complete SCI. The primary aim of this Phase I study was to assess the safety of the SCS implant, considering its off-label usage, in three patients with chronic motor complete SCI. Our secondary aims were to evaluate the synergistic potential of SCS and robotic neurorehabilitation in restoring volitional motor activity and achieving further clinical improvements, such as enhanced autonomic functions and independent locomotion. To meet these objectives, three patients were recruited to undergo customized SCS programming alongside a perioperative locomotor rehabilitation program.

Initially approved by the US FDA for chronic pain management [2,3], SCS has since evolved as a promising intervention for modulating neuronal activity in SCI [4,5,6]. Despite the therapeutic potential of combining SCS with advanced rehabilitation techniques, patient recruitment remains a significant challenge. SCI patients and their caregivers face considerable physical and emotional burdens, making participation in clinical trials particularly demanding. These difficulties are exacerbated by the need to balance safety and resource management, especially for an intervention like SCS, which involves invasive procedures and long-term follow-up. Moreover, strict eligibility criteria—while essential for ensuring study integrity—often lead to high screening failure rates, reducing the number of eligible participants and extending recruitment timelines [7]. Budgetary constraints and patient apprehension, especially in first-in-man trials, further complicate the recruitment process [7,8].

Understanding and addressing these barriers is essential for ensuring the success of this and similar trials in this patient population, as well as advancing therapeutic developments for SCI. Overcoming these multifaceted associated challenges are key to the trial’s success. Ensuring informed consent, patient compliance, and adherence to ethical recruitment practices are critical components that will be further explored in this paper.

## 2. Methods

### 2.1. Addressing the Limited Patient Population and Strict Eligibility Criteria

The annual incidence of chronic motor complete SCI is approximately 23 cases per million population [9]. This low incidence rate results in a relatively small pool of potential candidates for clinical trials, particularly those requiring stringent inclusion criteria. For the RESTORES trial [1], the highly specific eligibility requirements—targeting patients with chronic, motor complete SCI—was critical to ensuring patient safety and scientific validity of the trial. However, this also further limited the number of eligible participants. However, these strict criteria further constrained the pool of eligible participants, creating significant recruitment challenges.

To address these barriers, the study team implemented a multifaceted approach to maximize recruitment efforts. Central to this strategy was collaboration with multiple clinical centers specializing in SCI treatment and rehabilitation across Singapore [7]. Leveraging on the country’s well-integrated rehabilitation network, clear inclusion and exclusion criteria (Appendix A) were shared with referring clinicians, enabling them to identify and pre-screen suitable candidates effectively. These partnerships not only expanded the trial’s geographic and clinical reach but also reduced the likelihood of unnecessary disqualifications by aligning screening processes with trial requirements. Referring clinicians played a pivotal role in streamlining the recruitment pipeline by ensuring that only candidates meeting the eligibility criteria were referred for further assessment.

Additionally, the trial team actively engaged with national and regional SCI organizations, including patient support groups and rehabilitation centers, to enhance awareness and outreach. These organizations proved instrumental in connecting with potential participants and disseminating trial information through diverse channels, including print media, social platforms, and community events (Appendix B). By providing a trusted conduit for communication, these partnerships amplified the trial’s visibility within the SCI community and fostered a sense of inclusion and trust.

Together, these strategies effectively addressed the challenge of a limited candidate pool by broadening the recruitment network and ensuring a more focused and efficient screening process. This collaborative, multi-channel approach underscores the importance of leveraging clinical and community partnerships to overcome recruitment barriers in trials involving rare and complex conditions like chronic motor complete SCI.

### 2.2. Mitigating Patient Skepticism and Fear of Invasive Procedures

Patients may exhibit significant hesitancy toward participating in clinical trials involving spinal cord stimulation (SCS) due to concerns about the invasive nature of the procedure and its long-term management demands. These apprehensions can be further compounded by the off-label use of SCS, which may amplify doubts about its safety and efficacy. Recruiting patients for neurorestorative clinical trials has historically been challenging—not only in SCI but across conditions involving central nervous system (CNS) damage—primarily due to the long-standing belief that functional recovery in CNS injuries is impossible [10]. This widespread skepticism has deeply influenced both patients and clinicians’ attitudes. However, the field has seen rapid and consistent progress over the years [10,11].

To address these challenges, the RESTORES trial team implemented a comprehensive recruitment strategy centered on robust pre-surgical counselling designed to educate both patients and their caregivers. Each potential participant engaged in a minimum of three personalized, one-on-one counselling sessions with the principal investigator (PI) before enrolment. These sessions provided detailed insights into the SCS procedure, including its mechanism of action, safety profile, and outcomes from prior research. Importantly, patients were informed about global advances in neurorestoratology, including results from high-profile studies like E-STAND [12], ONWARD [13], and Up-LIFT [13], as well as yearly progress captured in the 2022 and 2023 Yearbooks of Neurorestoratology—discussing advances in neuromodulation, neurostimulation, and neurorestorative therapy [10,11]. Highlighting such emerging evidence helped counteract prior perceptions and offer patients tangible proof of ongoing clinical successes, which positively influenced their willingness to participate. Additionally, the discussions offered a platform for participants and their families to voice individual concerns, clarify uncertainties, and understand the potential risks, benefits, and expected outcomes of this study. By fostering open communication, the counselling sessions aimed to dispel misconceptions, alleviate fears, and build trust in the research team and procedures, thereby empowering informed decision-making.

Recognizing the significance of rigorous oversight in first-in-man trials, the study team established a data safety monitoring board (DSMB). This independent body, comprising a chairperson and four voting members unaffiliated with the trial’s conduct, played a pivotal role in addressing patient skepticism and ensuring this study’s integrity. The DSMB’s responsibilities included reviewing cumulative data to evaluate participant safety, assessing the trial’s scientific validity, and monitoring compliance with ethical standards. Before enrolling each participant, the DSMB meticulously examined their eligibility and analyzed safety data from previous participants to ensure continuous risk assessment.

By implementing an independent oversight mechanism, the DSMB not only reduced concerns about potential bias or oversight of adverse events but also bolstered this study’s credibility. Its proactive involvement reassured participants and caregivers that their safety and well-being were paramount. This commitment to transparency and accountability reinforced participant confidence, contributing significantly to overcoming recruitment barriers and fostering acceptance of this innovative, invasive therapeutic intervention.

### 2.3. Reducing Potential Medical Complications

Mechanical unloading, a frequent consequence of paralysis, induces rapid and severe osteoporosis in individuals with spinal cord injury (SCI), significantly increasing their susceptibility to fractures. This condition is primarily driven by the elevated expression of sclerostin by osteocytes, which inhibits bone formation, alongside indirect stimulation of bone resorption [14,15]. These effects are exacerbated by the loss of weight-bearing activities and hormonal dysregulation, further accelerating bone density loss [14,15]. Addressing this critical issue was essential for the RESTORES trial, as the rehabilitation regimen involved repetitive physical activities that could pose significant fracture risks in participants with compromised bone health.

To mitigate these risks, stringent screening protocols were implemented to exclude individuals with severe osteoporosis, defined by a T-score less than −2.5. Potential participants underwent comprehensive bone mass density (BMD) evaluations to ensure they met the safety criteria. For individuals with BMD scores indicating osteopenia (T-score between −1 and −2.4), additional assessments were conducted to confirm their suitability for participation, ensuring their bone health was sufficient to tolerate the rehabilitation program without undue risk.

The trial’s rehabilitation program incorporated a combination of advanced technologies and conventional physiotherapy to maximize therapeutic outcomes while prioritizing patient safety. A central component was the use of a wearable robotic exoskeleton, the EksoGT (Ekso Bionics, Richmond, CA, USA). Each participant was custom-fitted with the “robotic” exoskeleton, which facilitated supported, repetitive movements to enhance motor function and improve range of motion “of the lower limb”. This was complemented by conventional physiotherapy, which included targeted stretching and strengthening exercises for the trunk and extremities. Supported standing using a standing frame was gradually introduced to improve verticalization tolerance, while electric arm and leg cycling promoted cardiovascular health and joint mobility.

This comprehensive and carefully tailored approach balanced the need for rigorous rehabilitation with the imperative of safeguarding participant well-being. By proactively addressing osteoporosis-related risks, the trial ensured that participants could safely engage in the demanding rehabilitation activities, maximizing the potential for functional improvements while minimizing the likelihood of adverse outcomes. This strategy underscores the importance of integrating thorough pre-participation assessments and individualized care in clinical trials targeting populations with complex medical needs like chronic SCI.

### 2.4. Overcoming Geographical and Logistical Barrier

The trial’s rigorous daily rehabilitation schedule, spanning seven months and involving frequent follow-up visits, posed significant logistical challenges for participants, particularly those with limited mobility, financial constraints, or both. To ensure accessibility and minimize barriers to participation, the study team implemented a range of support measures tailored to address these challenges comprehensively.

Singapore’s well-developed public transportation infrastructure, including buses, the Mass Rapid Transit (MRT) system, and taxis, provided a reliable, affordable, and accessible means for participants to travel to the trial site. These transport options ensured that even participants residing outside central areas could maintain their rehabilitation schedules with relative ease. To further alleviate financial strain, participants were reimbursed for travel expenses, regardless of whether they used public transport, taxis, or private vehicles. This proactive measure ensured that transportation costs did not discourage individuals from participating, particularly those from lower-income households.

In addition to leveraging public transport, the trial team established robust logistical support systems to enhance participant accessibility. Detailed transport guides were provided to help participants navigate their travel routes efficiently. Flexibility in scheduling rehabilitation sessions was also introduced, accommodating individual preferences and personal commitments. For participants facing severe mobility challenges, specialized arrangements were made, such as coordinating with transport services designed to assist individuals with disabilities. These services included wheelchair-accessible vehicles and door-to-door pick-up and drop-off options, significantly easing the logistical burden for participants with higher support needs.

By implementing these inclusive and participant-centric strategies, the trial team successfully expanded accessibility, enabling a more diverse range of participants to engage in this study. These measures not only facilitated adherence to the demanding rehabilitation protocol but also fostered a supportive and equitable environment, underscoring the team’s commitment to overcoming logistical barriers and ensuring the trial’s success. This approach highlights the critical role of logistical planning in clinical trials, particularly those involving complex and resource-intensive interventions, and serves as a model for future studies aiming to enhance accessibility and participant engagement.

### 2.5. Addressing Emotional and Psychological Barrier

Patients with chronic spinal cord injuries (SCIs) often contend with profound emotional and psychological challenges, including depression, anxiety, and a pervasive fear of disappointment [16]. These issues can significantly diminish their motivation to participate in clinical trials, particularly those involving experimental interventions like spinal cord stimulation (SCS). Additionally, the emotional burden of balancing high hopes with the inherent uncertainties of such trials can deter potential participants [17,18].

To address these challenges, the RESTORES trial team implemented a robust pre-enrolment assessment process to evaluate each candidate’s mental and emotional well-being. This process included administering the *Diagnostic and Statistical Manual of Mental Disorders* (DSM-5) assessment and a Quality of Life (QoL) questionnaire. These tools helped identify psychological issues such as depression, anxiety, or emotional instability that could interfere with trial engagement. Beyond clinical assessments, in-depth discussions with potential participants focused on understanding their expectations, coping mechanisms, and mental resilience, which included sharing examples from contemporary international trials [12,13,19] and yearly clinical achievements, as mentioned above [10,11]. These trials involve pharmacologic, neuromodulation, and rehabilitative combinations, underscoring global recognition of the urgent need for functional recovery options in SCI. Incorporating such developments during counselling may improve decision-making and address therapeutic uncertainties. These conversations ensured that candidates were emotionally prepared to handle the physical and psychological demands of the trial, fostering a foundation for successful participation.

Realistic and transparent communication formed a cornerstone of the recruitment process [16]. The trial team emphasized the experimental nature of the intervention, clearly outlining potential outcomes, risks, and limitations. By setting achievable expectations, participants were better equipped to cope with both successes and setbacks, reducing the emotional toll of unmet hopes [6,7]. Regular progress updates, including videos showcasing functional improvements, were shared with participants to maintain motivation, reinforce their commitment, and instill a sense of accomplishment.

Recognizing the critical role of social support, the trial encouraged family and caregiver involvement throughout the process [20]. Families or close friends were invited to participate in trial-related discussions, where they received detailed information about the intervention, potential risks, and expected outcomes. This inclusive approach not only strengthened participants’ emotional and logistical support systems but also empowered family members to actively contribute to the rehabilitation journey. By fostering a collaborative and transparent environment, the trial team successfully addressed participants’ psychological concerns, ensuring a positive and supportive experience for both patients and their families [20,21].

### 2.6. Study Dedicated Clinical Research Coordinator (CRC)

A dedicated CRC played a pivotal role in ensuring the success of trial recruitment and managing the ethical and regulatory aspects of this study. The CRC’s responsibility included screening potential participants, ensuring they met this study’s eligibility criteria and coordinating their enrolment. Beyond these recruitment duties, the CRC also oversaw the trial’s compliance with institutional review board (IRB)-approved protocols, and ensured adherence to patient confidentiality regulations, such as the Personal Data Protection Act (PDPA) [22]. They also managed all necessary documentation to maintain the trial’s integrity [22]. The CRC coordinated communication between the research team and the IRB, keeping study status regularly updated, and ensuring that any amendments to the study protocol or forms were promptly reviewed and approved. Furthermore, the CRC prepared the site for potential audits, ensuring that the investigator’s site files were meticulously maintained and up to date [22,23]. By managing these regulatory and administrative responsibilities, the CRC ensured this study remained in compliance with ethical standards and regulatory requirements, while also guaranteeing the reliability and validity of the data collected. In a complex study like this, where multiple disciplines are involved with regulatory and ethical considerations to manage, a CRC acts as a linchpin, connecting various elements of the study—from patient recruitment and consent to data collection and reporting—ensuring that the research is conducted smoothly with credible and ethically sound study outcomes [22,23].

## 3. Results

**Figure 1:**
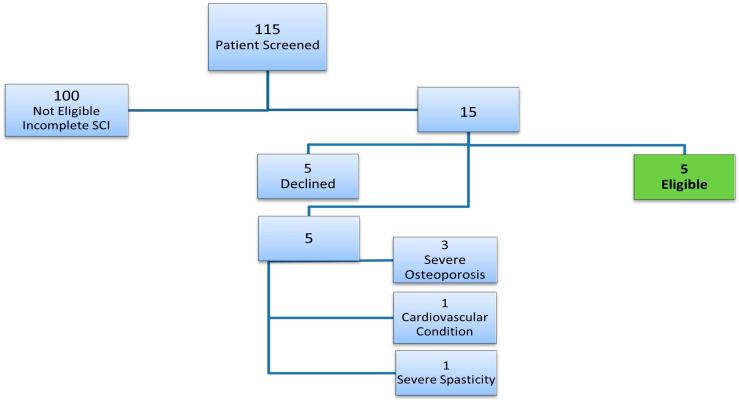
Patient Screening. Total number of patients screened, reasons for patient exclusion, and the final number of eligible patients.

During the recruitment period, 115 patients were screened for the RESTORES trial, as shown in Figure 1. Of these, 100 were deemed ineligible due to having incomplete spinal cord injuries (SCI), which did not meet the trial’s strict inclusion criteria. Among the remaining candidates, five patients declined participation, citing apprehensions about the intensive nature of the trial, which required a demanding rehabilitation schedule and invasive procedures. Additionally, three candidates were excluded due to severe osteoporosis, a condition that posed a significant risk for fractures during the rehabilitation regimen.

Two more patients were excluded based on specific criteria outlined in Appendix A—one due to a severe cardiovascular condition and the other because of significant spasticity that would interfere with the therapy protocol. Beyond medical factors, the participants’ level of determination and commitment emerged as a critical consideration. The trial required dedication to an intensive and prolonged therapy regimen, leading to the exclusion of two individuals who demonstrated insufficient motivation to adhere to the schedule.

Geographical and logistical challenges also impacted recruitment. Three patients residing outside Singapore expressed interest but were ultimately unable to participate due to the long-term commitment required and the need for frequent visits to the trial site. These logistical barriers, coupled with the trial’s rigorous demands, added to the overall recruitment challenges.

Collectively, these factors underscore the complexities of recruiting suitable candidates for a high-intensity, long-term clinical trial. They highlight the importance of addressing medical, logistical, and psychological barriers to participation, as well as the critical need for robust screening processes to ensure that participants are both medically eligible and fully prepared to commit to the demands of a study.

**Figure 2:**
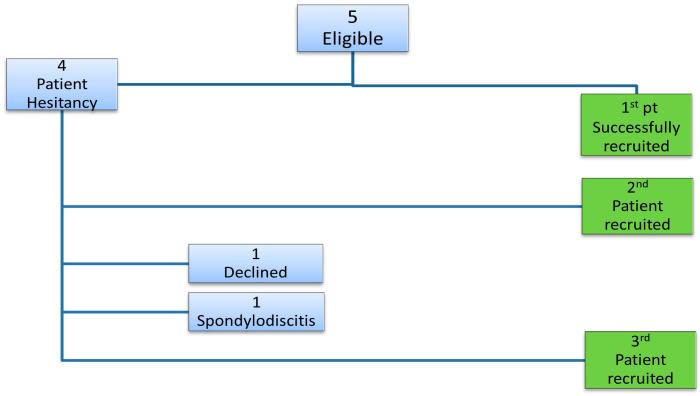
Patient selection. Recruitment process for the 5 eligible patients.

Recruitment for the third participant in our study presented significant challenges following the successful enrolment of the first two patients as shown in Figure 2. One potential participant withdrew after further consideration, citing personal reservations, while another promising candidate was disqualified due to a diagnosis of spondylodiscitis. Despite these setbacks, and after multiple screenings, we were able to recruit a final participant who met all inclusion and exclusion criteria.

Interestingly, the recruitment of the third participant was notably influenced by the encouragement of the first participant, underscoring the importance of peer support within the spinal cord injury (SCI) community. Both individuals were active members of an SCI peer support group, which fostered a strong sense of camaraderie and shared experience. The first participant, having achieved remarkable functional improvements in mobility and independence during the trial, became an advocate for this study. He shared detailed accounts of his experiences, including the procedural process and observed outcomes, which helped to address the concerns and build trust in the trial.

Motivated by the firsthand testimonial and the potential for similar benefits, the third participant chose to enroll in this study. This case highlights the significant role that peer influence and personal narratives can play in enhancing recruitment and fostering confidence in experimental treatments within patient communities.

## 4. Discussion

Spinal cord injury (SCI) is a significant global public health concern, affecting individuals across all ages and demographics [24]. While precise global prevalence rates are challenging to determine due to inconsistencies in data collection and reporting, estimates suggest that approximately 15 million people worldwide are living with SCI [25]. In Singapore, the annual incidence of SCI is reported to be 23 cases per million population [9,26], with a substantial proportion of cases attributed to traffic accidents [27]. This reflects global trends, where road traffic injuries are a leading cause of SCI [24,25]. A systematic review of SCI incidence in Asia reveals a wide variation, ranging from 10.8 to 22.7 cases per million population, underscoring the need for standardized data collection and reporting practices across the region [26].

The field of spinal cord injury (SCI) research is undergoing a transformative period, with emerging therapies and technologies offering new prospects for improved patient outcomes. Among these advancements, spinal cord stimulation (SCS) has emerged as one of the most promising interventions. Recent studies have demonstrated the potential of SCS to facilitate the restoration of neuronal networks and promote neurological recovery [17,19,28]. The RESTORES study represents a significant milestone in this field as the first-in-man trial conducted in Singapore. Designed as a feasibility study, RESTORES operated with limited resources and a small sample size, recruiting three participants to evaluate the project’s viability. This study aimed to systematically assess the technical feasibility of SCS, establish a standardized workflow encompassing all stages from patient recruitment to trial completion, and identify potential risks associated with the intervention.

Findings from this preliminary investigation provide critical insights to inform future efforts in refining and scaling this innovative therapeutic approach, laying the groundwork for broader clinical application and advancing the field of SCI rehabilitation.

### 4.1. Recruitment Barriers and Strategies

Recruitment for the RESTORES study posed several challenges, underscoring the complexity of enrolling suitable candidates for an intensive and pioneering trial. Key factors contributing to the exclusion of potential participants included the limited patient pool, skepticism about experimental treatments, stringent eligibility criteria, geographical and logistical barriers, and emotional and psychological concerns.

During initial consultations, emphasizing the importance of commitment proved critical in identifying motivated candidates who were likely to adhere to the rigorous therapy regimen [29]. This approach ensured that enrolled participants were well prepared for the demands of the trial.

The recruitment process revealed notable differences between self-referrals and professional referrals. Professional referrals, particularly those from physicians and physiotherapists, often yielded participants with higher eligibility consistency. This is likely attributable to the referrers’ comprehensive understanding of both this study’s inclusion and exclusion criteria and the patients’ medical conditions. Conversely, self-referred participants demonstrated greater self-motivation and higher levels of trust in the innovative nature of the intervention.

Collaboration with national and regional SCI organizations, including patient support groups and rehabilitation centers, further facilitated recruitment efforts by increasing awareness of this study. This strategy not only expanded the recruitment pool but also enabled connections with highly motivated individuals, such as members of SCI peer support groups. For instance, the recruitment of the third participant was influenced by the encouragement of the first participant, who had experienced significant functional improvements through the trial. By sharing his personal experience and insights, he alleviated the third participant’s concerns, built trust, and ultimately motivated her enrolment in this study.

Recent commentary by Scheuren et al. has urged the SCI field to embrace adaptive design features and pre-specified sub-group analyses to reduce ethical burden while preserving scientific validity [30]. While our trial employed strict eligibility criteria and pre-implantation screening, future studies may benefit from stratification models and registries to accelerate recruitment without compromising rigor.

These findings offer valuable insights for refining recruitment strategies, emphasizing the importance of leveraging both professional referrals and peer advocacy within the SCI community. Furthermore, the results underscore the critical role of personal testimonials and shared experiences in fostering patient confidence and participation in novel therapeutic trials.

### 4.2. Family Support

Spinal cord injury (SCI) is a life-altering condition that significantly diminishes an individual’s independence and quality of life. In most cases, caregiving responsibilities are undertaken by family members, often driven by social or economic factor [31]. Research has consistently highlighted the vital role of family support in the lives of individuals with SCI, with studies demonstrating that the absence of family caregivers increases the likelihood of patients being excluded from research studies [31]. Acknowledging this, the RESTORES study team actively encouraged the involvement of family members or close friends throughout this study. Caregivers were invited to participate in trial-related discussions, spanning from the pre-enrolment phase to study completion, fostering a sense of shared responsibility and support. This approach enabled caregivers to play an active role in the participant’s rehabilitation journey [20,21].

The RESTORES trial involved an intensive rehabilitation protocol, requiring a total commitment of seven months. Participants engaged in two sessions per week for one month prior to spinal cord stimulator (SCS) implantation and five sessions per week for six months post-implantation. The demanding schedule occasionally resulted in physical and mental fatigue, as well as fluctuations in mood among participants. While the multidisciplinary team provided professional support, the involvement of family members remained a cornerstone of care throughout the trial. Family caregivers played a critical role, not only by assisting participants with rehabilitation exercises at home but also by offering emotional stability, motivation, and a sense of belonging [18,32]. The study team prioritized transparent communication with participants and their families, providing regular updates on trial goals, including video documentation of functional improvements. This approach helped maintain engagement and optimism among participants [6,7]. The commitment of family members proved invaluable in sustaining participants’ motivation and adherence to the rehabilitation process. Their support was instrumental in facilitating recovery and ensuring that participants remained engaged and resilient throughout the trial [18].

Understanding that rehabilitation is not merely supportive but is an essential, synergistic partner in SCS-based recovery is crucial. As Boccia et al. noted, rehabilitation must be both personalized and accessible to meet the long-term needs of SCI patients [33]. Our approach integrates cost-effective, technology-supported rehabilitation to maximize patient outcomes, which in turn reduces the burden on both patients and their families.

### 4.3. Ethical Considerations

Ethical considerations were integral to every aspect of the trial, guided by the core principles of medical ethics: autonomy, beneficence, non-maleficence, and justice [34,35].

**Autonomy** was upheld by ensuring participants provided informed consent after a thorough understanding of the risks and benefits of this study [34,36]. Trial participants in our study underwent a comprehensive process that included counselling sessions with the PI prior to obtaining consent. This included detailed conversations about the study procedures, potential outcomes, and any associated risk. This approach ensured that patients’ decisions were based on their complete understanding of this study.
**Beneficence** was reflected in the trial design, which aimed to maximize potential benefits, such as improving neurological function through innovative therapies [36]. On top of that, the team ensured that participants were emotionally healthy and had strong family support to assist them throughout the trial before recruiting them.
**Non-maleficence** was prioritized by meticulously assessing risks, particularly given the invasive nature of spinal cord stimulation (SCS), and by maintaining participant safety as the trial’s foremost concern [34]. The strict eligibility criteria (Appendix A) established in our study were crucial in upholding the principle of non-maleficence, as we excluded patients suffering from severe spasticity and osteoporosis, safeguarding them against potential deterioration of their conditions.
**Justice** was addressed by promoting equitable access, including providing travel support and minimizing financial barriers, ensuring that all eligible participants, regardless of socioeconomic background, had an equal opportunity to participate [34,36].

The clinical research coordinator (CRC) played a pivotal role in managing these ethical and logistical challenges. Acting as the primary liaison between participants, the clinical team, and regulatory bodies, the CRC ensured that participants understood the trial procedures, addressed their concerns, and coordinated essential logistics, such as travel and follow-up care. Furthermore, the CRC maintained compliance with institutional review board (IRB) protocols, managed patient confidentiality, and oversaw the ethical and regulatory aspects of this study. By managing these critical responsibilities, the CRC ensured that recruitment and trial processes proceeded smoothly, ethically, and with a steadfast focus on participant welfare and this study’s integrity.

## 5. Conclusions

In conclusion, the RESTORES trial successfully addressed multiple recruitment barriers through a combination of strategic outreach, patient education, logistical support, psychological care, and the dedicated efforts of a CRC. These strategies were essential for overcoming the limited patient pool, managing patient apprehensions, navigating eligibility criteria, and addressing logistical and emotional challenges. This study highlights the necessity of innovative approaches and multidisciplinary collaboration in addressing the complex needs of SCI patients. The insights gained from this trial will inform future research and clinical practices, aiming to enhance therapeutic strategies and improve the overall quality of life for individuals living with chronic motor complete SCI.

## Figures and Tables

**Figure A1:**
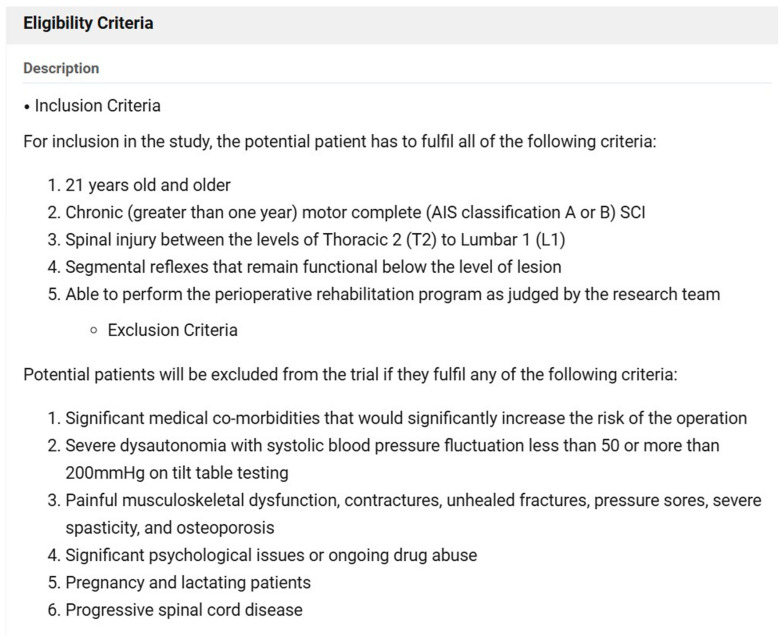
RESTORES study’s inclusion/exclusion criteria [37].

**Figure A2:**
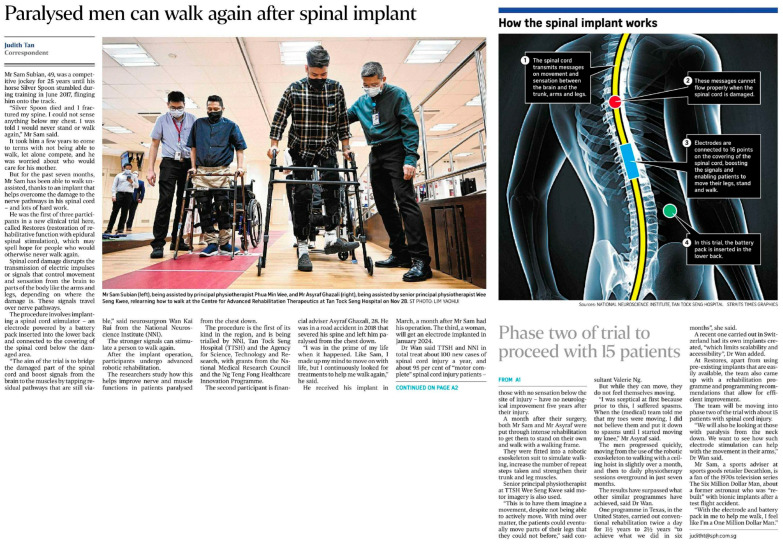

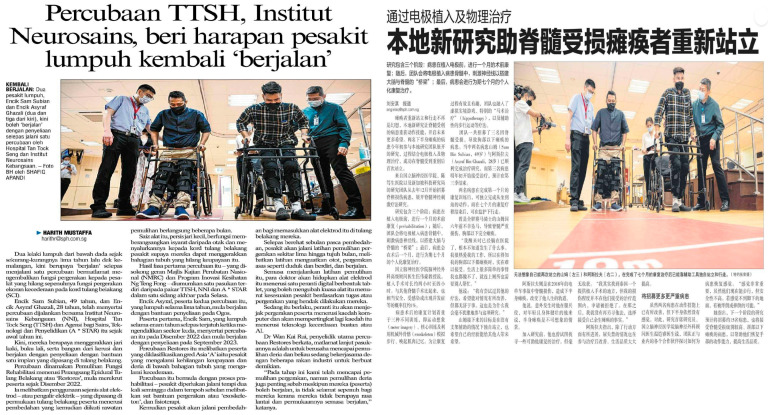
Singapore’s local newspaper, ‘The Straits Times’ article on the RESTORES study [38]. The same article was published, as seen below, in both the local Malay-language newspaper, Berita Harian, [39] and Chinese-language newspaper, Lianhe Zaobao [40].

## Data Availability

No new data were created or analyzed in this study. Data sharing is not applicable to this article.
